# Value of the application of computed tomography‐based radiomics for preoperative prediction of unfavorable pathology in initial bladder cancer

**DOI:** 10.1002/cam4.6225

**Published:** 2023-07-11

**Authors:** Situ Xiong, Wentao Dong, Zhikang Deng, Ming Jiang, Sheng Li, Bing Hu, Xiaoqiang Liu, Luyao Chen, Songhui Xu, Bing Fan, Bin Fu

**Affiliations:** ^1^ Department of Urology The First Affiliated Hospital of Nanchang University Nanchang China; ^2^ Jiangxi Institute of Urology Nanchang China; ^3^ Department of Radiology Jiangxi Provincial People's Hospital, The First Affiliated Hospital of Nanchang Medical College Nanchang China; ^4^ Department of Nuclear Medicine, Jiangxi Provincial People's Hospital The First Affiliated Hospital of Nanchang Medical College Nanchang China

**Keywords:** clinical model, initial bladder cancer, nomogram, radiomics, unfavorable pathology

## Abstract

**Objectives:**

To construct and validate unfavorable pathology (UFP) prediction models for patients with the first diagnosis of bladder cancer (initial BLCA) and to compare the comprehensive predictive performance of these models.

**Materials and Methods:**

A total of 105 patients with initial BLCA were included and randomly enrolled into the training and testing cohorts in a 7:3 ratio. The clinical model was constructed using independent UFP‐risk factors determined by multivariate logistic regression (LR) analysis in the training cohort. Radiomics features were extracted from manually segmented regions of interest in computed tomography (CT) images. The optimal CT‐based radiomics features to predict UFP were determined by the optimal feature filter and the least absolute shrinkage and selection operator algorithm. The radiomics model consist with the optimal features was constructed by the best of the six machine learning filters. The clinic‐radiomics model combined the clinical and radiomics models via LR. The area under the curve (AUC), accuracy, sensitivity, specificity, positive and negative predictive value, calibration curve and decision curve analysis were used to evaluate the predictive performance of the models.

**Results:**

Patients in the UFP group had a significantly older age (69.61 vs. 63.93 years, *p* = 0.034), lager tumor size (45.7% vs. 11.1%, *p* = 0.002) and higher neutrophil to lymphocyte ratio (NLR; 2.76 vs. 2.33, *p* = 0.017) than favorable pathologic group in the training cohort. Tumor size (OR, 6.02; 95% CI, 1.50–24.10; *p* = 0.011) and NLR (OR, 1.50; 95% CI, 1.05–2.16; *p* = 0.026) were identified as independent predictive factors for UFP, and the clinical model was constructed using these factors. The LR classifier with the best AUC (0.817, the testing cohorts) was used to construct the radiomics model based on the optimal radiomics features. Finally, the clinic‐radiomics model was developed by combining the clinical and radiomics models using LR. After comparison, the clinic‐radiomics model had the best performance in comprehensive predictive efficacy (accuracy = 0.750, AUC = 0.817, the testing cohorts) and clinical net benefit among UFP‐prediction models, while the clinical model (accuracy = 0.625, AUC = 0.742, the testing cohorts) was the worst.

**Conclusion:**

Our study demonstrates that the clinic‐radiomics model exhibits the best predictive efficacy and clinical net benefit for predicting UFP in initial BLCA compared with the clinical and radiomics model. The integration of radiomics features significantly improves the comprehensive performance of the clinical model.

## 1 INTRODUCTION

Bladder cancer (BLCA) is among the most commonly diagnosed malignant tumors of the urinary system, ranking as the tenth most prevalent cancer worldwide.^1^ The latest statistics indicate that BLCA caused approximately 210,000 tumor‐related deaths and affected 570,000 new individuals in 2020, posing a significant threat to public health.^1^ The pathology features of initial BLCA play a crucial role in determining the appropriate treatment strategy and predicting prognosis.^2^ Even though cystoscopic biopsies and transurethral resection of bladder tumor (TURBT) are standard methods for determining the histopathology of bladder tumors, up to 20%–53% of muscle‐invasive bladder cancers (MIBCs) are misdiagnosed and 15% of tumors are inaccurately graded due to sampling errors, heterogeneous characteristics of the tumor, and the small size of the sample collected.^3^, ^4^, ^5^, ^6^, ^7^ The standard of care for bladder tumors with unfavorable pathologic (UFP) features, such as high‐grade muscular infiltration, is radical cystectomy, whereas the first choice of treatment for bladder tumors with favorable pathologic (FP) feature, such as low‐grade nonmuscular infiltration, is TURBT.^8^, ^9^, ^10^ Inaccurate assessment of pathologic features can lead to incorrect treatment planning, increases the risk of poor outcomes for patients and causing significant financial and emotional damage.

Computed tomography (CT) images are the most commonly used method for preoperative evaluation of patients with BLCA. The morphology and enhancement pattern of CT can reflect certain pathological features of the bladder tumor. However, due to the limited number of features that the human eye can recognize from thousands of pixels in CT images, the information that can be acquired is limited. In contrast, the use of computer‐recognized imaging features can assist radiologists in making more accurate diagnoses.^11^ With the advancement of medical and computer technology, radiomics has gradually gained importance in urologic tumors.^12^, ^13^, ^14^ Radiomics is a new quantitative evaluation approach that involves converting digital medical images (including CT, magnetic resonance imaging (MRI), ultrasound, or positron emission tomography (PET) images) into high‐dimensional data and then extracting numerous image features through high‐throughput computation. CT‐based radiomics features of BLCA have been applied to predict the physiological information of tumors, such as preoperative histological grading and staging,^15^, ^16^, ^17^ achieving satisfactory predictive accuracy. However, most studies have only constructed predictive nomogram models containing imaging features, without incorporating additional omics information.

This study aimed to construct and validate three prediction models for UFP in initial BLCA, including the clinical, radiomics, and clinic‐radiomics models. The study further aimed to compare the comprehensive predictive performance of these models to identify the optimal model for aiding clinical treatment and prognostic assessment.

## 2 MATERIALS AND METHODS

### 2.1 Patient cohort

A total of 105 patients with initial BLCA diagnosed between 2017 and 2022 were included in the study and randomly enrolled into the training and testing cohorts, in a 7:3 ratio. Subjects were excluded according to the exclusion criteria: (1) missing clinical or imaging data; (2) incomplete clinical and imaging data at the time of initial diagnosis; (3) lack of pathological data after the CT scans conducted at our center; (4) history of any significant interventions such as radiotherapy and biopsy prior to CT scan.

### 2.2 Clinical research materials

Clinical‐pathological data, including years, sex, tumor size, number of tumors, smoking history, gross hematuria, neutrophil to lymphocyte ratio (NLR), histological grade and pathological stage, was extracted from a prospectively managed medical database. UFP was defined by the presence of high‐grade histology, BLCA with T stage ≥T2, BLCA with lymphatic metastasis (N1 stage) or BLCA with distant metastasis (M1 stage).

### 2.3 Clinical model construction

Univariable and multivariable logistic regression (LR) analyses were conducted on clinical characteristics to identify the independent predictive factors of UFP in the training cohort. These independent factors were then used to construct the clinical model (clinical UFP‐risk nomogram), and the clinical score was driven by Equation (1).

### 2.4 Imaging research materials

CT images of the abdomen or pelvic cavity were acquired using a SOMATOM Definition CT scanner. Scan parameters were automatically modulated according to the following settings: tube voltage, 120 kV; tube current, 150 mAs; slice thickness, 5 mm; reconstruction interval, 1 mm; and slice gap, 1 mm.

### 2.5 Region‐of‐interest (ROI) segmentation

The DARWIN scientific research platform (Beijing Yizhun Intelligent Technology Co., LTD., https://arxiv.org/abs/2009.00908) was utilized to delineate ROIs on the uploaded CT images. A radiologist (WD) with 5 years of experience manually segmented the 3D‐ROI, and another senior radiologist (BF) with over 15 years of experience reviewed all segmentation results. Both radiologists were blinded to the tumors' pathological features and the patients' assigned groups. In cases where there was a disagreement over the tumor segmentation boundary, the tumor profile was discussed and determined by the highly experienced radiologist.

### 2.6 Radiomics feature extraction and selection

After segmentation, 1781 radiomic features of lesions were extracted in the same platform using the Python Pyradiomics package (https://pyradiomics.readthedocs.io/en/latest/). The radiomic features included first‐order features, shape features, and texture features, such as gray‐level co‐occurrence matrix features, gray‐level run‐length matrix features, gray‐level size zone matrix features, and gray‐level dependence matrix features. Moreover, the radiomic features also included the first‐order features and texture features obtained from the images processed using nine filter types (original, exponential, gradient, lbp, logarithm, square, square root, wavelet, and log‐sigma). All radiomic features were normalized by *Z*‐score.

**FIGURE 1:**
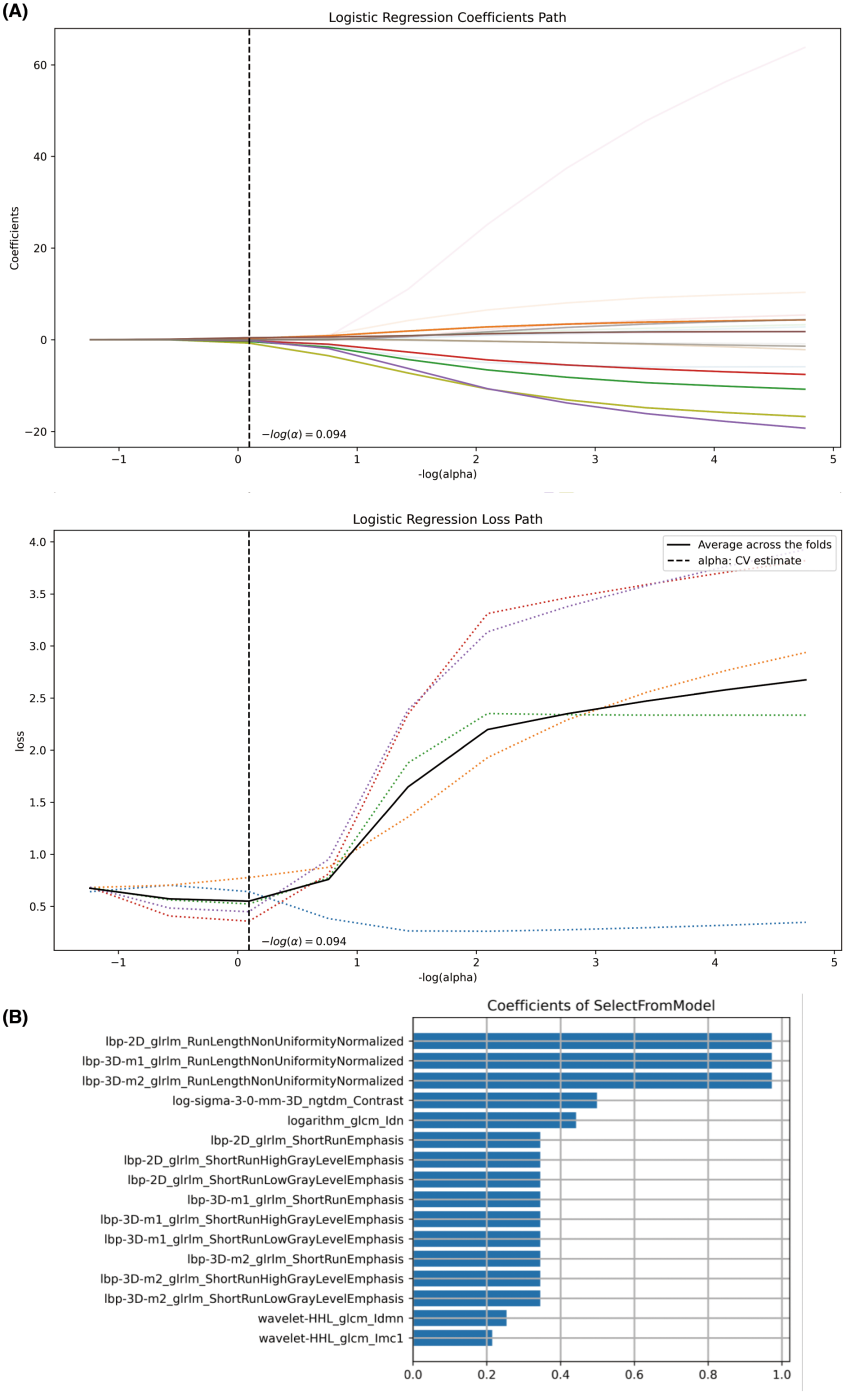
The optimal radiomic features selection (B) for UFP of initial BLCA based on LASSO regression algorithm (A). BLCA, bladder cancer; UFP, unfavorable pathology; LASSO, least absolute shrinkage and selection operator.

To determine the most relevant features, we evaluated the linear correlation between each feature and the category labels using the optimal feature filter, which was determined by the sample variance *F*‐value in the training cohort. Among the 1781 features, we filtered the 45 most relevant features were filtered. We then used least absolute shrinkage and selection operator regression to further select the best predictive features (Figure 1A). Finally, we extracted the 16 texture features that were most strongly associated with unfavorable pathology (UFP) in initial BLCA (Figure 1B).

### 2.7 Radiomics model construction

Based on the optimal radiomics features, we constructed six machine learning models in the training cohort, including gradient augmented decision tree (GBDT), K‐nearest neighbor, LR, random forest (RF), support vector machine, and EXtreme gradient boosting (XGBOOT). To confirm the accuracy of the selected model, we employed the 10‐fold cross‐validation method. The classifier with the highest predictive performance was selected to construct the radiomics model, and the radiomics score was obtained using Equation (1).

### 2.8 Clinic‐radiomics model construction

We developed the clinic‐radiomics model (clinic‐radiomics UFP‐risk nomogram) by combining the clinical and radiomics models using LR, which enabled us to determine the risk score with Equation (1):

(1) $$\text{Modelcore}=\alpha_{0}+{\alpha_{1}}^{*}x_{1}+{\alpha_{2}}^{*}x_{2}+\ldots +{\alpha_{n}}^{*}x_{n}$$

Here, *α*_*0*_ stands for the constant, *α*_*i*_ represents the LR coefficients, and *x*_*i*_ represents the value of chosen radiomics or clinical features. When constructing the clinic‐radiomics model, *x*_*i*_ denotes the score of each individual model.

### 2.9 Model performance evaluation

**FIGURE 2:**
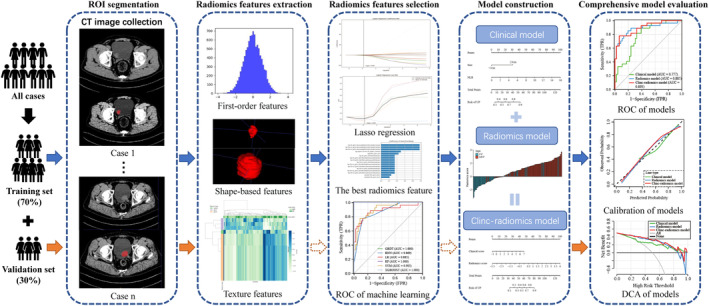
Workflow of this study. The dotted arrow indicates that this step is skipped.

To assess the comprehensive predictive performance of the models in both the training and testing cohorts, we quantified the area under the curve (AUC) of the receiver operating characteristic, as well as the accuracy (ACC), sensitivity (SEN), specificity (SPE), positive predictive value (PPV) and negative predictive value (NPV). We employed DeLong's test to compare the differences in AUC between the models. Calibration curves were employed to evaluate the calibration of the models, and decision curve analyses (DCA) were applied to gauge the clinical net benefit, which can indicate the clinical utility of the models. The workflow of this study was demonstrated in Figure 2.

### 2.10 Statistical analysis

All categorical variables compared with Pearson's chi‐squared test were given in the form of numbers and percentages. The Student *t*‐test was used to assess normally distributed continuous variables, which were reported as mean and standard deviation; for those with non‐normal distribution, the Wilcoxon rank sum test was used, and their results were presented as median and interquartile range. SPSS 24.0 software (SPSS) and R software (version 4.1.0) were utilized for all statistical analyses and graphical representations. All tests were two‐sided, and significance was set at *p* < 0.05.

## 3 RESULTS

### 3.1 Clinic‐pathological characteristics

Ultimately, 27 patients with PF and 46 patients with UFP were included to the training set, while 12 patients with PF and 20 patients with UFP were included in the testing set. Table 1 showed the clinicopathological characteristics of the patients. Patients in the UFP group had a significantly older age (69.61 vs. 63.93 years, *p* = 0.034), lager tumor size (45.7% vs. 11.1%, *p* = 0.002) and higher NLR (2.76 vs. 2.33, *p* = 0.017) than the FP group in the training cohort. No statistically significant differences were found between the groups in regard to other variables (*p* > 0.05). Furthermore, no significant differences were observed between the training and testing cohorts (*p* > 0.05).

**TABLE 1:** Demographic and clinical information between patients of FP and UFP cohort in the training and testing cohorts.

	Training cohort				Testing cohort	*p* value
	Total	FP	UFP	*p* value		
Patient, *n* (%)	73 (69.5)	27 (37.0)	46 (63.0)	NA	32 (30.5)	NA
Age, years, mean (SD)	67.51 (11.13)	63.93 (12.24)	69.61 (9.97)	**0.034**	68.16 (10.25)	0.779
Sex, *n* (%)				1		1
Female	11 (15.1)	4 (14.8)	7 (15.2)		5 (15.6)	
Male	62 (84.9)	23 (85.2)	39 (84.8)		27 (84.4)	
Tumor size, *n* (%)				**0.002**		0.286
≤3 cm	49 (67.1)	24 (88.9)	25 (54.3)		18 (56.3)	
>3 cm	32 (32.9)	3 (11.1)	21 (45.7)		14 (43.8)	
Number of tumors, *n* (%)				0.232		0.691
Single	52 (71.2)	17 (63.0)	35 (76.1)		24 (75.0)	
Multiple	21 (28.8)	10 (37.0)	11 (23.9)		8 (25.0)	
Smoking history, *n* (%)				0.832		0.295
Without	63 (86.3)	23 (85.2)	40 (87.0)		25 (78.1)	
With	10 (13.7)	4 (14.8)	6 (13.0)		7 (21.9)	
Gross hematuria, *n* (%)				0.847		1.000
Without	18 (24.7)	7 (25.9)	11 (23.9)		8 (25.0)	
With	55 (75.3)	20 (74.1)	35 (76.1)		24 (75.0)	
NLR, median (IQR)	2.62 (1.94–4.63)	2.33 (1.71–3.00)	2.76 (1.99–5.51)	**0.017**	2.71 (2.09–4.31)	0.536
Histological grade				NA		0.634
Low grade	31 (42.5)	27 (100)	4 (8.7%)		12 (37.5)	
High grade	42 (57.5)	0 (0.0%)	42 (91.3%)		20 (62.5)	
Pathological stage				NA		0.327
< T2	30 (39.7)	27 (100)	2 (4.3%)		16 (50)	
≥ T2	44 (60.3)	0 (0.0%)	44 (95.7%)		16 (50)	
Metastasis						
Lymphatic metastasis	3 (4.1%)	0 (0.0%)	3 (6.5%)	NA	1 (3.1%)	1.000
Distant metastasis	1 (1.4%)	0 (0.0%)	1 (2.2%)	NA	0 (0.0%)	1.000
Radiomics score, mean (SD)	0.73 (1.49)	−0.53 (1.31)	1.47 (1.03)	**<0.0001**	0.85 (1.35)	0.696

*Note*: Bolded numbers mean statistically different, that is *p* < 0.05.

Abbreviations: FP, favorable pathology; IQR, interquartile range; NA, not applicable; NLR, neutrophil to lymphocyte ratio; SD, standard deviation; TURBT, transurethral resection of bladder tumor; UFP, unfavorable pathology.

### 3.2 Construction of the three UFP‐prediction models

**FIGURE 3:**
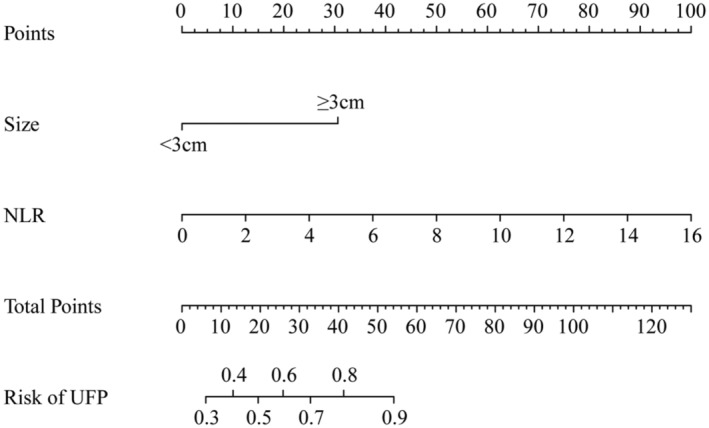
Clinical model (clinical UFP‐risk nomogram) for predicting the risk of UFP in patients with initial BLCA. BLCA, bladder cancer; UFP, unfavorable pathologic.

After conducting univariable and multivariable LR analyses in the training cohort (Table 2), tumor size (OR, 6.02; 95% CI, 1.50–24.10; *p* = 0.011) and NLR (OR, 1.50; 95% CI, 1.05–2.16; *p* = 0.026) were identified as independent predictive factors of UFP for initial BLCA. Subsequently, the clinical model (clinical UFP‐risk nomogram) was constructed based on the risk factors (Figure 3). The formula for the clinical model was provided in the supplementary material.

**TABLE 2:** Univariate and multivariate logistic regression analysis for independent predictive factors of UFP.

Variables	Univariate analysis		Multivariate analysis	
	Crude OR (95% CI)	*p*‐Value	Adjusted OR (95% CI)	*p*‐Value
Age	1.05 (1.00–1.10)	**0.040**	1.05 (0.99–1.11)	0.110
Gender				
Female (Re.) vs. male	0.97 (0.26–3.67)	0.963		
Tumor size				
≤ 3 cm (Re.) vs. > 3 cm	6.72 (1.77–25.49)	**0.005**	6.02 (1.50–24.10)	**0.011**
Number of tumors				
Single (Re.) vs. Multiple	0.53 (0.19–1.50)	0.235		
Smoking history, *n* (%)				
Without (Re.) vs. With	0.86 (0.22–3.38)	0.832		
Gross hematuria, *n* (%)				
Without (Re.) vs. With	1.11 (0.37–3.33)	0.847		
NLR, median (IQR)	1.49 (1.07–2.07)	**0.018**	1.50 (1.05–2.16)	**0.026**

*Note*: *p* value <0.05 indicates a significant difference and are bolded.

Abbreviations: NA, not applicable; TURBT, transurethral resection of bladder tumor; UFP, unfavorable pathology.

The prediction performances of six machine learning models were shown in Figure S1A,B. Comparison of the AUC in different models between the training and testing cohorts revealed falsely high predictive performance in the GBDT, RF, and XGBOOST classifiers in the training cohort. However, the LR classifier not only had good prediction performance in the training group (AUC = 0.885, ACC = 0.822, SEN = 0.852, SPE = 0.804, PPV = 0.719 and NPV = 0.902), but also had the best prediction performance (AUC = 0.817, ACC = 0.750, SEN = 0.700, SPE = 0.833, PPV = 0.875 and NPV = 0.625) than remaining classifiers in the testing cohort. Therefore, the LR classifier was chosen to construct the radiomic model. Figure S2A exhibited the distribution of the radiomics score for all cases, in which patients with UFP had a higher radiomics score than UP. In addition, as depicted in Figure S2B, the difference was statistically significant not only in the training cohort (*p* < 0.0001) but also in the testing cohort (*p* < 0.01) (Figure 3). The formula of radiomics model was provided in supplementary material.

**FIGURE 4:**
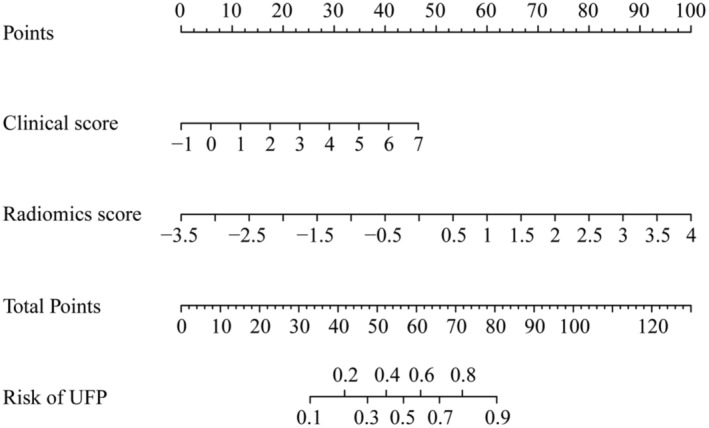
Clinic‐radiomics model (clinic‐radiomics UFP‐risk nomogram) for predicting the risk of UFP in patients with initial BLCA. UFP, unfavorable pathologic; BLCA, bladder cancer.

Similarly, the clinic‐radiomics model, known as the clinic‐radiomics UFP‐risk nomogram, was developed using LR analysis and by combining radiomics score with clinical score (Figure 4). To generate a UFP‐risk score for each case, the expression for the clinic‐radiomics score (outlined in the supplementary material) was utilized.

### 3.3 Validation and comparison of the three UFP‐prediction models

**FIGURE 5:**
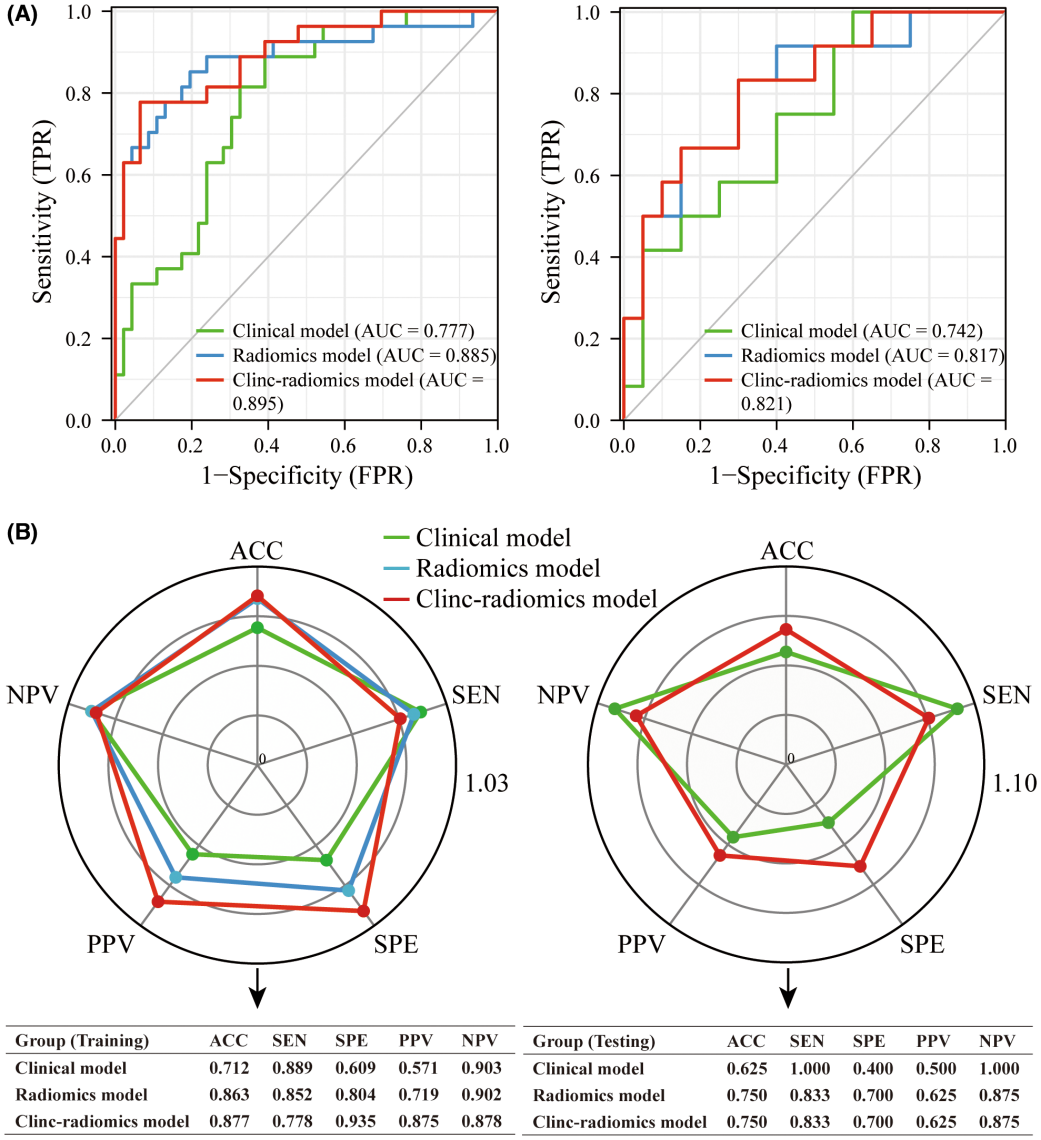
The predictive performance of three UFP‐prediction models, including clinical model, radiomics model and clinic‐radiomics model. ROC of three UFP‐prediction models in the training cohort (A, left) and testing cohort (A, right). The predictive performance of three UFP‐prediction models in ACC, SEN, SPE, PPV and NPV in the training cohort (B, left) and testing cohort (B, right). ACC, accuracy; AUC, area under the curve; NPV, negative predictive value; PPV, positive predictive value; SEN, sensitivity; SPE, specificity; UFP, unfavorable pathologic.

**FIGURE 6:**
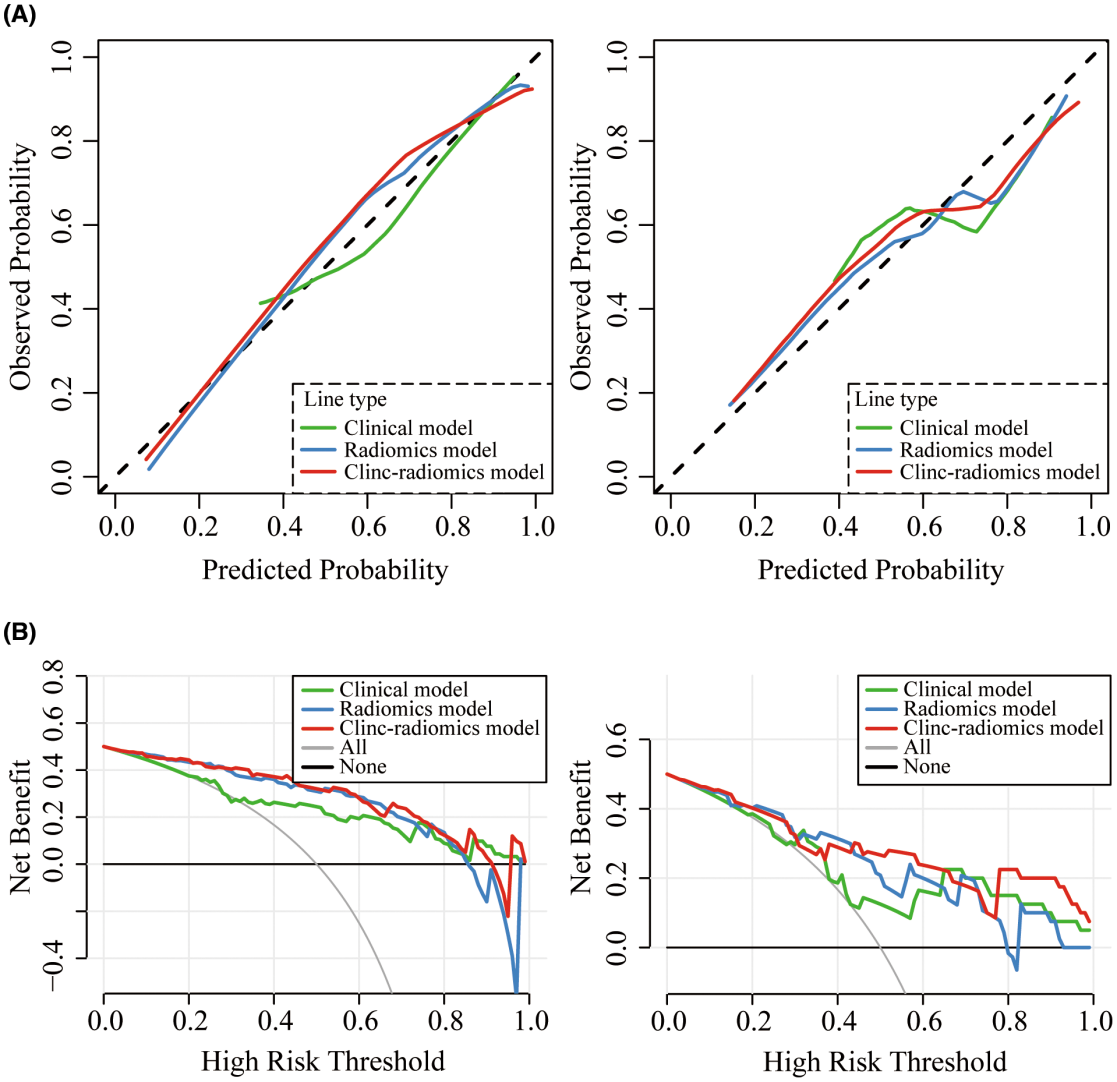
Calibration curve of three UFP‐prediction models in the training cohort (A, left) and testing cohort (A, right). DCA of three UFP‐prediction models in the training cohort (B, left) and testing cohort (B, right). DCA, decision curve analyses; UFP, unfavorable pathologic.

As shown in Figure 5A, the clinic‐radiomics model demonstrated the highest AUC value among the training cohort (AUC = 0.895, 95% CI = 0.818–0.973) and testing cohorts (AUC = 0.821, 95% CI = 0.669–0.972), surpassing both the clinical model and radiomics model. The results of DeLong's test (Table 3) indicated a statistically significant difference between the clinic‐radiomics model and the clinical model in the training cohort (*p* = 0.015), but not in any other intergroup comparisons (*p* > 0.05). Nevertheless, the clinic‐radiomics model was once again confirmed with better predictive performance for UFP in patients with initial BLCA by the comprehensive comparison of the ACC, SEN, SPE, PPV, and NPV values of the different UFP‐prediction models in the training (0.877, 0.778, 0.935, 0.875, and 0.878, respectively) and testing cohorts (0.750, 0.833, 0.700, 0.625 and 0.875, 0.750, 0.833, 0.700, 0.625, and 0.875) (Figure 5B). The calibration curves demonstrated good agreement between the actual observation and estimated probability for all three UFP‐prediction models in both cohorts (Figure 6A). Analysis of DCA curves in the training cohort (Figure 6B, left) revealed that when the high‐risk threshold was within a range of 0%–82.5%, using the radiomics model and clinic‐radiomics model to identify pathological features and make therapeutic decisions had greater clinical benefit than the clinical model, “treat all” and “treat none” schemes. In contrast, the clinical benefit of the radiomics model was not superior to that of the clinical model in the testing cohort (Figure 6B, right). Nevertheless, the clinic‐radiomics model still provided the best outcome among the three UFP‐prediction models.

**TABLE 3:** Comparison of the AUC with the clinical model, the radiomics model, and the clinic‐radiomics model.

Data set	Model 1	Model 2	*p* Value
Training	Clinical model	Radiomics model	0.082
	Radiomics model	Clinic‐radiomics model	0.546
	Clinic‐radiomics model	Clinical model	**0.015**
Testing	Clinical model	Radiomics model	0.415
	Radiomics model	Clinic‐radiomics model	0.809
	Clinic‐radiomics model	Clinical model	0.355

*Note*: *p* value <0.05 indicates a significant difference and are bolded.

## 4 DISCUSSION

In recent years, radiomics has become a popular method for preoperative prediction of bladder tumor pathology, thanks to advances in medical technology, and has achieved remarkable success in that regard.^12^, ^17^, ^18^, ^19^ Several researchers have applied CT, MRI, and ultrasound to radiomic studies for predicting histological grading and staging features of BLCA and have demonstrated good predictive efficacy.^20^, ^21^ However, in most studies, only predictive nomogram model that contained imaging features was constructed, and models for other histologies were not. Zhao et al. combined radiomic and preoperative clinical features of perihilar cholangiocarcinoma to form a clinic‐radiomics nomogram for forecasting early recurrence, which proved to be the most effective (AUC = 0.868) compared to the single‐element model.^22^ Therefore, we hypothesized that combining multi‐omics data could potentially further improve the performance of predictive models for BLCA. To test our hypothesis and assess the value of radiomics in predicting pathological features for initial BLCA by clinical characterizations, we constructed and validated three UFP‐prediction models which relied on the optimal clinical and radiomic features, including a clinical model, radiomics model, and clinic‐radiomics models. Overall, the clinic‐radiomics model demonstrated the highest predictive efficacy and clinical net benefit among the three models, while the clinical model lagged. Thus, we concluded that radiomics could significantly improve the performance of the clinical model, and recommended that urologists use the clinic‐radiomics model to examine the pathological features of patients with initial BLCA.

Clinical characteristics are widely used in the construction of nomogram models for preoperatively predicting the pathological features of various tumors; certainly, urologic oncology is no exception.^23^, ^24^ In this study, the clinical model of UFP for initial BLCA was constructed based on tumor size (OR, 6.02; 95% CI, 1.50–24.10; *p* = 0.011) and NLR (OR, 1.50; 95% CI, 1.05–2.16; *p* = 0.026). Several studies have pointed out that tumor size is significantly associated with adverse pathological features. Sylvester et al. and Fernandez‐Gomez et al. have both identified the size of the bladder tumor as a prognostic factor.^25^, ^26^ In addition, the EAU and NCCN guidelines use a tumor size of 3 cm as the threshold for risk classification.^10^, ^27^ Mariappan et al. found that the risk of incorrectly predicting high‐grade BLCA as low‐grade significantly decreased in larger tumors (>30 mm) (OR, 3.2; 95% CI, 1.0–9.7; *p* = 0.07).^28^ Moreover, increasing evidence in recent years has highlighted that higher NLR correlates with lower BLCA pathology, as well as worse tumor outcomes. The study by Viers et al. evaluated the correlation between preoperative NLR and clinicopathological outcomes following radical cystectomy in 899 cases, and revealed that with a 1‐unit increase of NLR, there was a 7% and 9% increased risk of UFP for extravesical tumor extension (*p* = 0.03) and lymph node involvement (*p* = 0.02), respectively.^29^ Similarly, the work of Tang et al. suggested that a higher NLR signified a higher risk of high‐grade and muscle‐invasive disease.^30^ Multiple studies have also corroborated the connection between NLR and oncologic outcome, and it has been utilized as a marker for risk evaluation.^31^, ^32^ The above demonstrates that the independent predictors used to construct the clinical model for UFP of initial BLCA are valid and reliable.

The present study revealed that the clinical model had AUC values of 0.777 and 0.742 in the training and testing cohorts, respectively, indicating its appropriateness for predicting UFP in patients with initial BLCA. Nevertheless, the radiomics model exhibited marginally better predictive efficacy in both the training group (AUC = 0.885 vs. 0.777) and the testing group (AUC = 0.817 vs. 0.742), although these differences did not reach statistical significance. However, compared to the clinic‐radiomic model, the difference in AUC was statistically significant in the training group (0.777 vs. 0.895, *p* = 0.015). These results demonstrated that the integration of radiomics into the clinical model led to an increase in the predictive efficacy of the model. This was reflected in a statistically significant difference in AUC between the clinic‐radiomic and clinic‐only models in the training group (0.777 vs. 0.895, respectively). The predictive accuracy and net benefit of the model were also found to be significantly improved with the inclusion of radiomics, further reinforcing that radiomics can lead to greater accuracy and overall performance when used in combination with a clinical model.

Radiomics features can reveal small, subtle changes in medical images that are difficult to detect with the unaided eye. These changes can help to characterize and interpret changes in tumor biology, providing timely and accurate information for clinical purposes.^33^ Among the radiomic features included in this study, local binary pattern (LBP) features account for the most significant proportion (12/16, 75%). The LBP filter can detect the image's microstructure by comparing local voxel gray levels and creatingbinary pattern codes, which has been successfully utilized in computer‐aided diagnosis systems for various malignant tumors.^34^, ^35^ Machine learning classifiers are essential for optimizing the performance of radiomics models in terms of accuracy and diagnosis. Their inclusion allows for enhanced assessment of medical images and more reliable outcomes.^36^ In this study, LR had the best classification performance (AUC, ACC, SEN, SPE, PPV, and NPV of 0.885, 0.822, 0.852, 0.804, 0.719, and 0.902, respectively) among six classifiers in both the training and testing cohort. LR is widely used in machine learning classifiers related to radiomics analysis because of its simplicity. The incorporation of regularization into the LR model helps to reduce the error between the predicted and actual values, while also moving the weights closer to their origins. This helps to prevent overfitting and enhances the model's generalization capabilities.^17^ Radiomics offers numerous benefits which lead to the overall predictive performance of the clinic‐radiomics model being much greater than that of the clinical model alone. These advantages allow for superior accuracy and greater overall performance when it comes to predicting outcomes.

Although this study is the first to construct three models and systematically compare their comprehensive performance in predicting pathological features in initial BLCA, our study is not without limitations. First, this study is retrospective in nature, so there is potential for selective bias, and further prospective research should be conducted to confirm the results. Second, since the data was drawn from a single medical center, the sample size was relatively small, further limiting the scope of the study. Multicenter studies with large sample sizes are still needed to reduce regional differences and to evaluate the models. Third, some biological features or biomarkers that are valuable for guiding clinical diagnosis and treatment are not included in the model, such as genetic mutation data. Incorporating additional genetic information into clinical features and thus constructing more comprehensive clinic‐radiomics predictive models may allow us to achieve higher predictive efficacy in future studies.

## 5 CONCLUSION

Our study constructed three models for predicting UFP in patients with initial BLCA. The multi‐omics model that combines CT based radiomics and clinical models had the best predictive efficacy and clinical net benefit for predicting UFP in initial BLCA. Radiomics contributed significantly to the improvement of the comprehensive performance of the clinical model.

## AUTHOR CONTRIBUTIONS

**Situ Xiong:** Conceptualization (equal); formal analysis (lead); methodology (equal); software (equal); visualization (equal); writing – original draft (lead); writing – review and editing (lead). **Wentao Dong:** Conceptualization (equal); data curation (equal); methodology (equal); software (equal); supervision (equal). **Zhikang Deng:** Data curation (equal); investigation (equal); software (equal); supervision (equal). **Ming Jiang:** Investigation (equal); supervision (equal); validation (equal). **sheng Li:** Investigation (equal); methodology (equal); project administration (equal). **Bin Hu:** Investigation (equal); methodology (equal); project administration (equal). **Xiaoqiang Liu:** Investigation (equal); methodology (equal); supervision (equal); funding acquisition (equal). **Luyao Chen:** Investigation (equal); methodology (equal); supervision (equal). **Songhui Xu:** Conceptualization (equal); investigation (equal); methodology (equal); project administration (equal); supervision (equal); funding acquisition (equal). **Bing Fan:** Conceptualization (equal); investigation (equal); methodology (equal); resources (lead). **Bin Fu:** Conceptualization (equal); funding acquisition (equal).

## FUNDING INFORMATION

This study was supported by the National Natural Science Foundation of P.R. China (Grant Nos. 81960512, 82172921).

## CONFLICT OF INTEREST STATEMENT

The authors declare that they have no competing interests.

## ETHICS STATEMENT

The study was approved by the Institutional Review Board of Jiangxi Provincial People's Hospital, and written informed consent was waived.

## Supporting information


Figure S1.

## Data Availability

Data supporting the results of this study may be obtained from the corresponding author upon reasonable request.
