# Supplementary material for: Value of the application of computed tomography‐based radiomics for preoperative prediction of unfavorable pathology in initial bladder cancer
Source: Cancer Med. 2023 Jul 11;12(15):15868–80. doi: 10.1002/cam4.6225 (PMC10469743; doi:10.1002/cam4.6225)
Supplement: Supplementary file 1 — Figure S1. [file CAM4-12-15868-s001.docx]

The formula of clinical model was as follows:

**Clinical score**＝-1.233789＋1.942872*Size＋0.396479*NLR

The formula of radiomics model was as follows:

**Radiomics score**=-0.729

-0.910*lbp-2D_glrlm_RunLengthNonUniformityNormalized

-0.910*lbp-3D-m1_glrlm_RunLengthNonUniformityNormalized

-0.910*lbp-3D-m2_glrlm_RunLengthNonUniformityNormalized

+0.510*log-sigma-3-0-mm-3D_ngtdm_Contrast

-0.434*logarithm_glcm_Idn

+0.334*lbp-2D_glrlm_ShortRunEmphasis

+0.334*lbp-2D_glrlm_ShortRunHighGrayLevelEmphasis

+0.334*lbp-2D_glrlm_ShortRunLowGrayLevelEmphasis

+0.334*lbp-3D-m1_glrlm_ShortRunEmphasis

+0.334*lbp-3D-m1_glrlm_ShortRunHighGrayLevelEmphasis

+0.334*lbp-3D-m1_glrlm_ShortRunLowGrayLevelEmphasis

+0.334*lbp-3D-m2_glrlm_ShortRunEmphasis

+0.334*lbp-3D-m2_glrlm_ShortRunHighGrayLevelEmphasis

+0.334*lbp-3D-m2_glrlm_ShortRunLowGrayLevelEmphasis

-0.269*wavelet-HHL_glcm_Idmn

-0.139*wavelet-HHL_glcm_Imc1

The formula of clinic-radiomic model was as follows:

**Clinic-radiomics score**＝-0.387914＋1.231119* Radiomics score＋0.536716* Clinical score


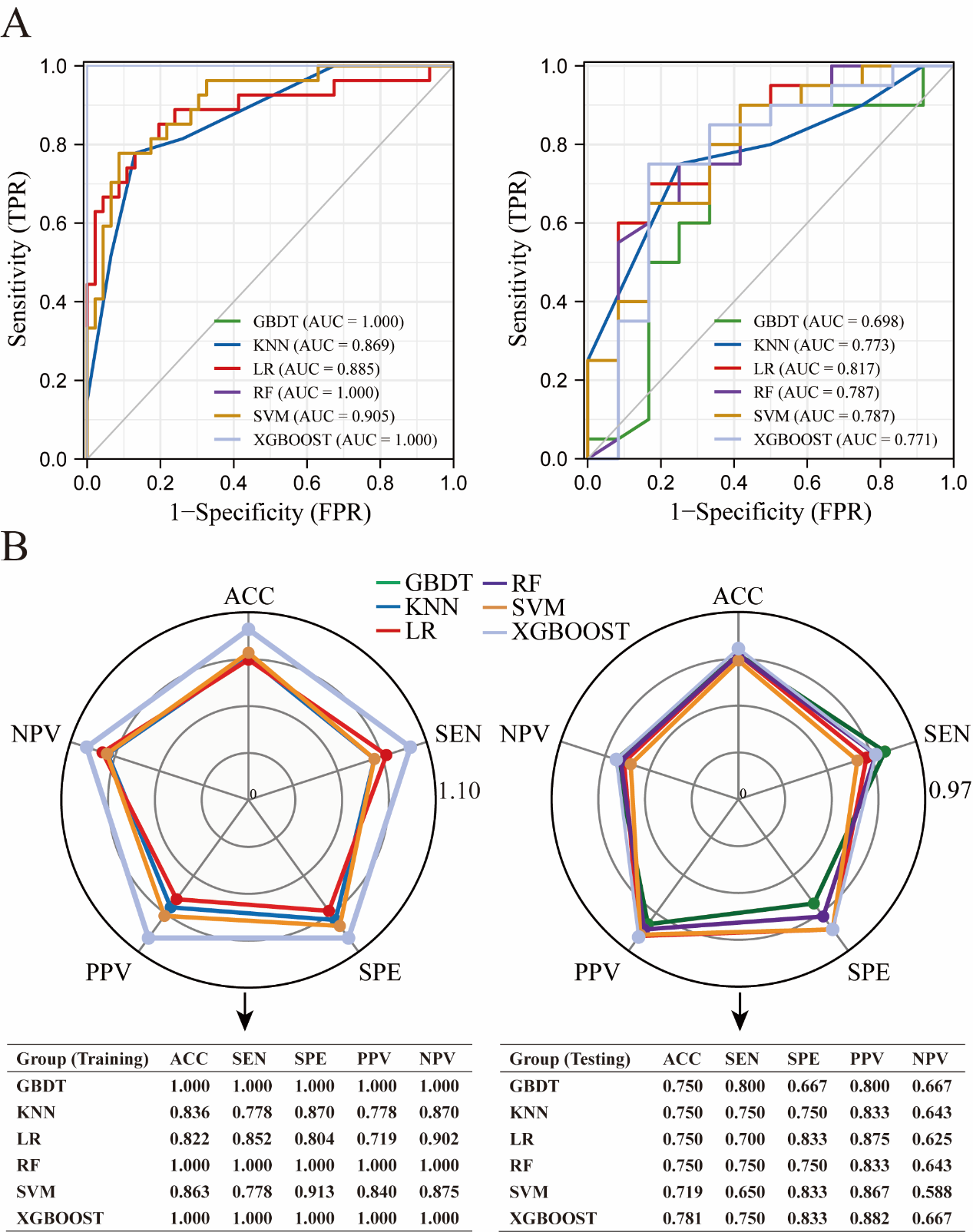
**Fig S1.** The predictive performance of six machine learning radiomics models, including GBDT, KNN, LR, RF, SVM, XGBOOST radiomics models. ROC of six machine learning radiomics models in the training cohort (A, left) and testing cohort (A, right). The predictive performance of six machine learning radiomics models in ACC, SEN, SPE, PPV and NPV in the training cohort (B, left) and testing cohort (B, right). GBDT, gradient boosted decision tree; KNN, k-Nearest Neighbor; LR, logistic regression; RF, random forest; SVM, support vector machine; XGBOOST, eXtreme Gradient Boosting; AUC, area under the curve; ACC, accuracy; SEN, sensitivity; SPE, specificity; PPV, positive predictive value; NPV, negative predictive value.


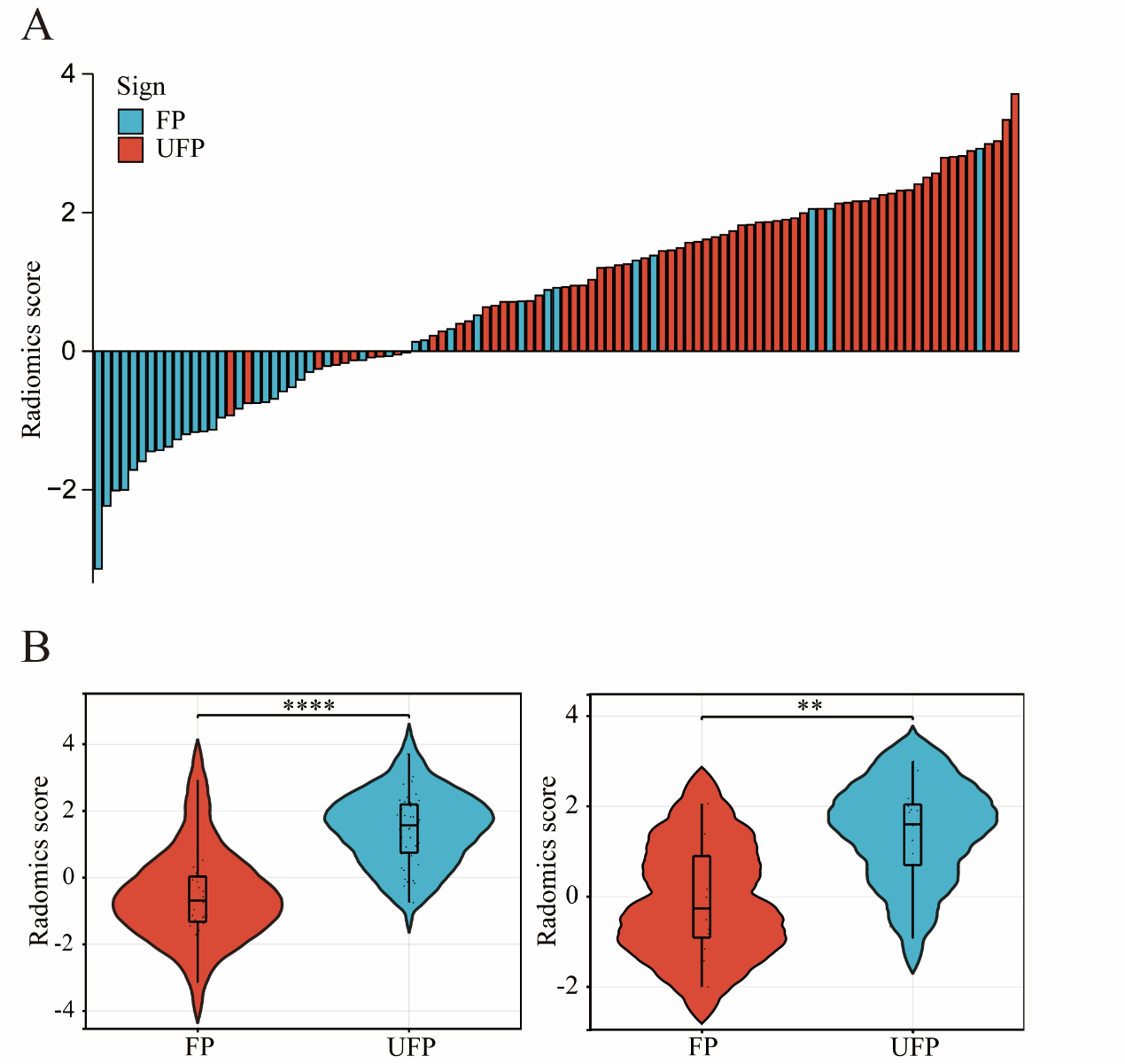
**Fig S2.** Radiomics score in all cases (A) and in the training cohort (B, left) and testing cohort (B, right). FP, favorable pathologic; UFP, unfavorable pathologic.

**, *P*<0.01; **** *P*<0.0001.
